# Perimenstrual Asthma in Adolescents: A Shared Condition in Pediatric and Gynecological Endocrinology

**DOI:** 10.3390/children9020233

**Published:** 2022-02-10

**Authors:** Valeria Calcaterra, Rossella Elena Nappi, Andrea Farolfi, Lara Tiranini, Virginia Rossi, Corrado Regalbuto, Gianvincenzo Zuccotti

**Affiliations:** 1 Pediatric and Adolescent Unit, Department of Internal Medicine and Therapeutics, University of Pavia, 27100 Pavia, Italy; 2 Department of Pediatrics, “Vittore Buzzi” Children’s Hospital, 20154 Milano, Italy; andrea@farolfi.it (A.F.); virginia.rossi@unimi.it (V.R.); gianvincenzo.zuccotti@unimi.it (G.Z.); 3 Research Center for Reproductive Medicine, Gynecological Endocrinology and Menopause, Fondazione IRCCS Policlinico San Matteo, 27100 Pavia, Italy; rossella.nappi@unipv.it (R.E.N.); lara.tiranini01@universitadipavia.it (L.T.); 4 Department of Clinical, Surgical, Diagnostic and Pediatric Sciences, University of Pavia, 27100 Pavia, Italy; 5 Pediatric Unit, Fondazione IRCCS Policlinico San Matteo, University of Pavia, 27100 Pavia, Italy; corrado.regalbuto01@universitadipavia.it; 6 Department of Biomedical and Clinical Science “L. Sacco”, University of Milano, 20157 Milano, Italy

**Keywords:** perimenstrual asthma, sex hormones, adolescents, menstrual cycle

## Abstract

Asthma is a frequent medical condition in adolescence. The worsening of the most common symptoms perimenstrually is defined as perimenstrual asthma (PMA). The cause of PMA remains unclear, but a role for hormonal milieu is plausible. Data on PMA in adolescents are limited, and its management is not fully established. We aimed to discuss the PMA phenomenon in young females from pathophysiology to preventive strategies, focusing on the relationship with the hormonal pattern. The fluctuation of estrogens at ovulation and before menstruation and the progesterone secretion during the luteal phase and its subsequent withdrawal seem to be the culprits, because the deterioration of asthma is cyclical during the luteal phase and/or during the first days of the menstrual cycle. Conventional asthma therapies are not always effective for PMA. Preventive strategies may include innovative hormonal contraception. Even a possible beneficial effect of other hormonal treatments, including estrogens, progestogens, and androgens, as well as leukotriene receptor antagonists and explorative approach using microbial-directed therapy, is considered. The underlying mechanisms, through which sex-hormone fluctuations influence asthma symptoms, represent a challenge in the clinical management of such a distressing condition. Further studies focused on young females are mandatory to promote adolescent health.

## 1. Introduction

Asthma is one of the commonest noncommunicable diseases; it is a frequent medical condition in childhood and adolescence, characterized by high prevalence (approximately 5–10%), chronic nature, potentially severe symptoms, and associated burden on healthcare resources [1,2].

The global prevalence of asthma is characterized by wide variability among countries: it is highest in developed countries and lowest in emerging ones; nevertheless, its burden is increasing rapidly in developing countries as lifestyles become more Westernized [3,4]. Nearly 25 million people in the United States have asthma (approximately 300 million people globally) [5]. According to the CDC (Centers for Disease Control and Prevention), its prevalence ranges from 9.1 to 9.7% in adult women and 5.1 to 5.5% in men [6].

Asthma is characterized by respiratory symptoms, such as coughing, wheezing, shortness of breath, chest tightness, and enhanced mucus production, that vary over time. It is usually associated with variable airflow limitation and hyper-responsiveness at lung function testing, and with markers of airways inflammation in some patients [2]. Asthma can worsen during the perimenstrual period, which is an event known as perimenstrual asthma (PMA). The cause of PMA remains unclear, but a role for hormonal milieu is plausible. Fluctuation of estrogens at ovulation and before periods, along with progesterone secretion during the luteal phase and its subsequent withdrawal, seem to be the culprits, because deterioration of asthma is cyclical during the luteal phase and/or during the first days of the menstrual cycle.

PMA occurs in up to nearly 40% of asthmatic women of reproductive age [7]. The beneficial effects of hormonal treatments, as well as leukotriene receptor antagonists and microbial-directed treatment strategies, have been proposed [8,9,10,11,12,13,14,15,16,17]. Data on PMA in adolescents are limited, and its management is not fully established. 

In this paper, we aim to discuss the PMA phenomenon in young females from pathophysiology to preventive strategies, focusing on the possible relationship with the hormonal pattern. The underlying mechanisms through which fluctuations of sex hormones influence asthma symptoms represent a challenge in the clinical management of such distressing condition. 

## 2. Methods

We performed a search for English journal articles published in the past 15 years up to October 2021, following the criteria of a narrative review [18]. V.R., C.R., and L.T. independently identified the most relevant published studies, including original papers, meta-analysis, clinical trials, and reviews. Case reports, series, or letters were excluded. The following keywords (alone or in combination) were used: “perimenstrual asthma”, “adolescents”, “menstrual cycle”, “hormones”, “hormonal pattern”, “females”, and “sex hormone”. The electronic databases PubMed, Scopus, EMBASE, and Web of Science were searched. The contributions were critically reviewed by V.C., R.E.N., and A.F. The resulting draft was discussed with all co-authors. The final version was then recirculated and approved by all.

## 3. Hormonal Pattern of Menstrual Cycles

The activation of the hypothalamic–pituitary–gonadal (HPG) axis occurs at puberty, a developmental stage marked by maturation of the gonads, secretion of sex steroid gonadal hormones (namely estradiol and progesterone), and appearance of secondary sexual characteristics [19]. Changes in the pulsatile secretion of the hypothalamic gonadotropin-releasing hormone (GnRH) trigger the release of the pituitary gonadotropins follicle-stimulating hormone (FSH) and luteinizing hormone (LH). In turn, gonadotropins stimulate ovarian follicle maturation with the subsequent gradual increase of circulating gonadal sex steroids. Estradiol induces proliferative changes of the endometrium, followed by secretory changes induced by progesterone, thus leading to menstruation at the time of hormonal withdrawal [20]. 

Menstrual cycle consists of several phases, starting from the first day of menstrual bleeding (day 1). Briefly, in the follicular phase, among a cohort of primordial follicles under the influence of FSH, the dominant one matures in about 10–12 days and secretes increasing amount of estradiol. When estradiol reaches its peaks into the circulation, a positive feedback mechanism induces the pituitary secretion of LH into the bloodstream, and ovulation occurs 36 h after, around the 13th or 14th day of the menstrual cycle. Following ovulation, luteinized granulosa and theca cells form the corpus luteum, releasing progesterone and estradiol during the luteal phase. If conception does not take place, luteolysis occurs with consequent marked decline in both sex hormones, a trigger for menstruation. While the duration of luteal phase is notably constant (12–14 days), the length of follicular phase can vary according to different follicle maturation time [20]. 

It must be considered that the HPG axis extensively interacts with other neuroendocrine axes, and its relationship with the hypothalamic–pituitary–adrenal (HPA) axis is extremely relevant for female fertility [21]. Even androgens manifest fluctuations during the menstrual cycle and exert several influences upon the normal functioning of the HPG axis. Androgenic milieu is the result of both ovarian and adrenal cortex secretion, along with the contribution of peripheral tissues. Indeed, androstenedione is produced half by the ovary and half by the adrenal gland, and its serum levels increase around ovulation. Testosterone, the most potent androgen, is synthetized by the ovary and the adrenal glands and results from the peripheral conversion of androstenedione. It reflects the same menstrual variations of androstenedione, with higher levels at mid-cycle and during the luteal phase compared with the early follicular phase [22]. Dehydroepiandrosterone (DHEA) and dehydroepiandrosterone sulfate (DHEA-S) are weak androgens deriving mostly from the adrenal glands and manifest a circadian rhythm as cortisol, the principal product of the HPA axis. Overall, androgens influence ovarian and menstrual functions through their conversion into estrogens, the consequent feedback on pituitary release of gonadotropins, the enhanced production of progesterone from the follicular cells, and the inhibition of granulosa cells proliferation leading to follicular atresia [23].

There is a general consensus that, in the first 1 or 2 years after menarche, the majority of menstrual cycles are anovulatory and characterized by heterogeneous hormonal profiles, different stages of follicle development, and exposure to unopposed estrogens, thus resulting in menstrual cycles with irregular pattern of length [24,25,26]. While advancing gynecological age, functions of HPG axis progress first to ovulatory cycles with luteal insufficiency and then to mature ovulations, thus enabling regular menstrual patterns (25–35 days) and reproductive potentials by late adolescence [24,25]. Since irregular menstruations have been related to an increased risk of asthma in adult women [27,28,29,30], this association should be kept in mind and further explored also in young girls.

**Figure 1:**
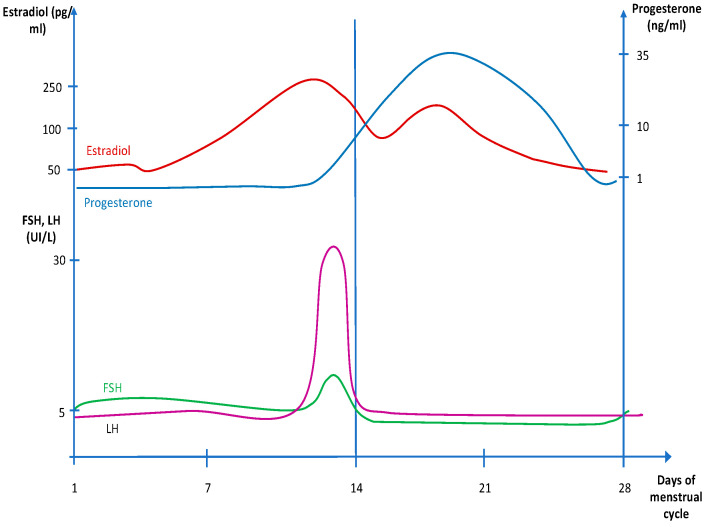
Fluctuations of sex hormones during the menstrual cycle.

The normal hormonal values [31] and fluctuations according to the phases of the menstrual cycle are reported in Table 1 and Figure 1, respectively. 

## 4. Asthma in Young Female Adolescents

Globally, in people aged 10 to 24 years, respiratory disorders are the sixth leading cause of disability; in 2011, it was estimated that asthma was responsible for 346,000 deaths worldwide each year [32,33]. In pediatric age, the prevalence of asthma is higher in males than in females; however, in adulthood, the prevalence is approximately 20% higher in females than in males, indicating a change that occurs during puberty [3,34]. The higher prevalence in boys is, in part due, to their smaller airways relative to lung size compared with young girls; however, this is a characteristic that reverses during adolescence [3]. 

The Global Initiative for Asthma (GINA) guidelines describe asthma as a heterogeneous disease [2], usually characterized by chronic airway inflammation [1]. Variability in symptoms and airflow limitation is a feature of asthma that can vary in time and intensity [2]. Clusters of demographic, clinical, and pathophysiologic characteristics identify asthma phenotypes and endotypes. No strong relationship has been found between specific pathological features and peculiar clinical patterns and treatment responses; however, in patients with more severe asthma, some phenotype guided treatments have become available [2,35,36,37]. 

Epidemiologic evidences have shown that the overall prevalence and incidence of asthma are increased in obese individuals, and obesity is a risk factor for airway inflammation [32,38,39,40,41,42]. A recent meta-analysis, including six prospective cohort studies on the effect of body weight on future asthma risk, found a twice-higher risk in obese children compared with normal-weight ones, suggesting that obesity is an independent asthma risk factor for the youngest [43,44]. Clinical studies also suggest that obesity-related asthma is distinct from normal-weight asthma: it is associated to decreased responsiveness to medications [45] and poor disease control [42,43,46], particularly among ethnic-minority children [46,47], contributing to increased healthcare expenditures [43]. In terms of ethnic and gender differences, it has been observed that Hispanics and African Americans, who have a higher burden of obesity-related asthma, tend to have central obesity more frequently, for the same body weight, than Caucasians [43,48], and obese girls are more symptomatic [49,50] than boys [51]. Visceral obesity is responsible for a picture of mechanical obstruction in the lungs, resulting in airflow obstruction and altered lung volumes [43]. The relationship between obesity and asthma is complex [38,52]. In the Severe Asthma Research Program, an age/phenotype-dependent association was found: children with early onset asthma became obese, whereas there was no significant relationship between overweight/obesity and asthma duration in cases of late-onset asthma [32,53]. In the Asthma Adiposity Study conducted by Kattan et al. [47] among 368 adolescents aged 12 to 20 years living in urban areas of the United States, the main finding was the association between adiposity and asthma morbidity [47]. In female adolescents, higher BMI and body fat correlated with worse asthma control, more disease exacerbations, and a lower Tiffenau index (forced expiration, FEV1/forced vital capacity, FVC) [47]. In addition, obese girls, in contrast to boys, lacked the anti-inflammatory properties of serum adiponectin. Indeed, although there were higher serum levels of adiponectin in females, its protective role was observed only in males: one plausible interpretation is that adiponectin receptors are downregulated with elevated adiposity in female adolescents [47]. Adipokines produced by the adipose tissue are likely to mediate the association between obesity and asthma in a gender-specific manner, and asthma outcome seems to be adversely influenced by obesity in girls but not in boys [32,47]. 

Despite a worldwide reduction in asthma mortality in adults and children over the past 25 years, largely attributable to increased use of inhaled corticosteroids, a wide global disparity in life years lost due to asthma remains [3].

It is much easier to diagnose asthma in teenagers than in younger children. A narrower range of conditions should be considered in the differential diagnosis of the adolescent who presents with recurrent cough, dyspnea, or wheezing [54]. In contrast to preschoolers, it is possible to assess lung function at baseline and in response to bronchodilators or exercise for objective evidence of reversible airway narrowing. However, both under-diagnosis and under-treatment are common [54,55]. Of note, in a Danish study, it has been shown that girls were 50% more likely to have underdiagnosed asthma than boys (69% vs. 33%), and coughing rather than wheezing or breathlessness was the major symptom [56].

Remission of asthma frequently occurs, especially in late adolescence, with reported rates of 16% to 60% [57]. In several population-based studies, remission probability of asthmatic disease in late adolescence was commonly observed and showed a relationship with mild disease, male sex, and absence of atopic trait, particularly sensitization to fur-bearing animals at the age of 7 or 8 years [32,58]. In a prospective study conducted in Sweden since 1996 in people followed from 7 or 8 to 19 years of age, 21% of those with asthma at age 7 were in remission at 19 years old, 38% had periodic asthma, and 41% had persistent asthma [58]; these results were similar to those of other studies [57,59,60,61,62]. Conversely, sensitization and female sex have been identified as important predictors of persistent asthma in several studies [59,63] In addition, along with a higher incidence among girls [64], the higher remission rate among boys contributes to the change in asthma prevalence ratio between boys and girls. That notwithstanding, puberty is the turning point with male prevalence in early childhood and a more prevalent asthma among women later in life [58]. Other relevant risk factors for pubertal asthma include early airway obstruction, sensitization to fur-bearing animals, more severe asthma in childhood, family history of atopy, being the first-born child, perinatal family stress, extreme preterm birth (23–27 gestational weeks), and low birth weight per gestational age [32,58,63,65,66,67,68]. In young females, early menarche (before 12 years of age) has been associated with an up to two-fold increased risk of new onset asthma [30,69]. However, the role of heredity, sex, smoking, and sensitization to specific allergens remains to be further investigated [32,70,71,72,73,74].

As far as the role of sex is concerned, both incidence and prevalence of asthma vary accordingly; in addition, age-related changes in sex prevalence are observed [32,75]. Sex differences play a key role in driving numerous conditions, including cardiovascular diseases and, specifically, atherosclerosis [76]; bone metabolism disorders, such as osteoporosis [77]; and some neurological pathologies [78]. Sex hormones are responsible for the expression of such gender differences at the phenotypic and genotypic level [79], including the regulation of airway function and inflammation [79]. Indeed, it has been observed that estrogens are able to prevent cholinergic constriction of asthmatic tracheal rings in vitro [80], and estrogen treatment decreases airway responsiveness to acetylcholine in ovariectomized rats [81]. Female rats appear to be more susceptible than males to allergen-induced airway inflammation [82,83,84]. By examining sex differences in a scenario of allergen exposure delivered exclusively through the airway and in the absence of systemic sensitization and adjuvant, thus dependent solely on IgE, Fc epsilon receptor (FceRI), and mast cells [79], the inhibitory effects of estrogen on airway hyper-responsiveness (AHR) development are evident [80,81,85,86]. In contrast, when dual allergen sensitization with systemic adjuvant and airway sensitization occurs, female mice develop greater AHR, with estrogen increasing airway inflammation [83,84,87]. Carey et al. also demonstrated that estrogen receptor deficiency led to increased AHR [85].

Unquestionably, female sex hormones, particularly estrogens, play a key role in the pathophysiology of asthma and the development AHR. 

Estrogens participate in various biological processes through different molecular actions. They may show either pro- or anti-inflammatory properties depending on the circumstances and the involved tissues. 

Lowered levels of estrogens increase the concentration of reactive oxygen species, along with the inhibition of NF-KB transcription factor, indicating their prevalent anti-inflammatory properties.

On the other hand, estrogen therapy in ovariectomyzed mice reduces reactive oxygen species-induced by ovariectomy in bone marrow [88,89], attenuating the phosphorylation of PKC β (redox-sensitive cytoplasmic kinase) [88,89].

It is well-known that female patients of all ages endure remarkably lower rates of infection and resultant mortality than male subjects. 

Immunological evidences suggest that female sex hormones are key factors in the etiology and course of chronic inflammatory diseases, being linked to significant influencing reproductive stages, such as menstrual cycle, pregnancy, perimenopause, and postmenopausal status [90,91,92,93]. Estrogens and their specific receptors influence systemic immune response by reducing the cytokine-driven cortisol and ACTH release, by increasing substance P signaling and responsiveness to noxious stimulation (increase of neurogenic inflammation).

Estrogen receptors α and β could have different effects depending on the cell type. The connection between estrogens and their receptors acts on monocyte differentiation along with inflammatory mediator production by macrophages [94,95,96]. Estrogens, in particular, 17 β-estradiol (E2), protect neuronal cells against toxic insults, inducing the expression of growth factors and their receptors, as well as proteins and cells involved in apoptosis [94,95,96]. 

The prevalence of asthma is higher in males before puberty, with a reversal in adulthood, when it is more common in women, who have a higher risk of having severe asthma, as well as greater susceptibility to the harmful effects of smoking [32,97,98,99]. There is no single direct mechanism responsible for gender differences, but several etiologies have been proposed, including gender-specific dysinaptic lung growth, female and male hormonal influences, genetic susceptibility, immune response, and differences in consultation practices [32,85,100,101,102]. Studies showing changes in lung structure and function at key life stages, such as puberty, suggest a modulatory role of sex steroids in the phenomenon of asthma transitions [32]. Indeed, sex hormones are known to have both biological and pathophysiological actions on several non-reproductive organs, including the lung [103]. Moreover, pulmonary function appears to undergo alterations during the menstrual cycle, with worsening of asthma when sex hormones decline premenstrually [104,105]. Moreover, a number of women who have moderate asthma report relief of their premenstrual exacerbations by taking oral contraceptives, which suppress wide fluctuations in circulating sex hormones [102]. Although these preliminary clinical data seem to indicate that the hormonal milieu correlates with prevalence and severity of asthma, the role of specific hormones in determining these changes is unclear. In particular, the effects of estrogens in asthma have been discordant [83,84,85]. On one side, supplementation with estrogens has been used beneficially as sparing steroids in women with asthma. Conversely, postmenopausal estrogen therapy increased the subsequent risk of asthma, and several lines of evidence report worsening of asthma during the premenstrual and intra-menstrual periods [74,104,106,107,108,109].

Even progesterone may play a role in the exacerbation of asthma symptoms [110]. Indeed, progesterone can influence airway smooth muscle tone and inflammation. Moreover, a progestogen hypersensitivity, characterized by a spectrum of symptoms ranging from urticaria to asthma and systemic anaphylaxis, has been described [110].

In addition, from a therapeutic point of view, there is evidence suggesting that the response to inhaled corticosteroid may be gender-specific: in particular, women are less likely to have uncontrolled persistent asthma [111]; the underlying rational is currently not understood [32,112].

Besides the intrinsic risk factors mentioned above, extrinsic risk factors, such as environmental pollution and cigarette smoking, also play a major role on asthma incidence and severity [113,114,115]. Furthermore, females seem to have a greater susceptibility to the effects of smoking [32], which, whether occasional or not, is a serious risk factor for adolescents with asthma, and smoking rates in this vulnerable population remain high [32,116]. Early exposure to secondhand smoke, whether in utero or during childhood, influences the development of allergic disease into adolescence [117]. Guerra et al., who conducted a prospective study from birth to age 26, recently demonstrated that active and parental smoking act synergistically to influence early deficits in lung function in young adulthood [118]. In addition, in asthmatic individuals, tobacco smoking is associated with accelerated decline in lung function, decreased disease control, and reduced benefit from corticosteroid treatment [119]. Although electronic cigarettes (e-cigs), as compared to traditional ones, contain fewer carcinogens and cause fewer acute lung effects in both healthy individuals and asthmatics, they are still not totally harmless, as they contain formaldehyde and other toxins that are still poorly understood [120,121]. Moreover, e-cigs could offer an alternative to cannabis use for youth [32,122]. However, due to the absence of complete scientific studies related to cannabinoid vaping, health consequences remain largely unknown and hypothetical; therefore, the most significant health concerns are for children and adolescents [122]. In addition, smoking cannabinoids could lead to environmental and passive contamination [122].

Adolescence is a period fraught with expectation for the youngest, in which social, sexual, and intellectual maturation occurs, followed by a progressive greater level of autonomy [123]. Nevertheless, this autonomy is frustrated by parental and physician dependence for asthma care, medication, and the need for continued follow-up [123]. Asthma, and how it is managed, can impede these processes and intensify the stress that is already an integral part of ordinary adolescence [54].

Anxiety and depression are clinically linked to asthma in this life period [124,125,126], being typically associated with lower adherence to daily monitoring of asthma symptoms [125,127,128]. Social anxiety disorder (SAD), one of the most common anxiety disorders in adolescents, is typically characterized by intense fear of social situations, anxiety, or avoidance; all of which lead to significant impairment (e.g., few friends, loneliness, depressed mood, poor school performance, and difficulty with interpersonal relationships) [128,129]. SAD can be particularly disabling during adolescence because peers play a critical role in social and identity development at this stage [128,130]. Adolescents with asthma are then at high risk for social anxiety. These youth report feeling different and isolated from their peers, fearing peer rejection and having poor social competence [129,131,132,133,134]. In addition, social anxiety may decrease compliance with treatment regimens that require taking medications in front of others (e.g., taking rapid-release medications before exercise or exposure to other triggers), because adolescents are worried about being accepted by peers [128]. Overall, anxiety disorders, such as panic disorder (PD), are up to twice as prevalent in females as in males [135], and it appears that a gender-specific susceptibility is responsible for their development. Sex hormonal changes occurring in the premenstrual phase represent a neuromodulatory signal in the onset and maintenance of maladaptive or clinical anxiety and other mental disorders in the female sex [136,137,138,139,140]. Indeed, in some [107,141,142] but not in all [104] studies, women with PMA showed a higher incidence of premenstrual syndrome if compared with a control asthma group [143]. Dysphoric symptoms or general discomfort preceding menstruation [141] could contribute to self-reported perimenstrual worsening of asthma: women with PMS might have an altered perception of asthmatic symptoms in the premenstrual phase, and emotional changes may influence lung function up to precipitation of asthmatic attacks [143]. In a study conducted by Richardson et al. [125], involving subjects aged 11–17 years, youth with an anxiety or depressive disorder were, among other things, more frequently girls and had a more recent diagnosis of asthma. Furthermore, in the same research by Richardson et al. [125], youth with an anxiety or depressive disorder reported, on average, more days of symptoms than the other participants without either of these disorders (5.4 vs. 3.5 days). Among other factors significantly and independently associated with increased symptom days, there was the female sex. 

**Figure 2:**
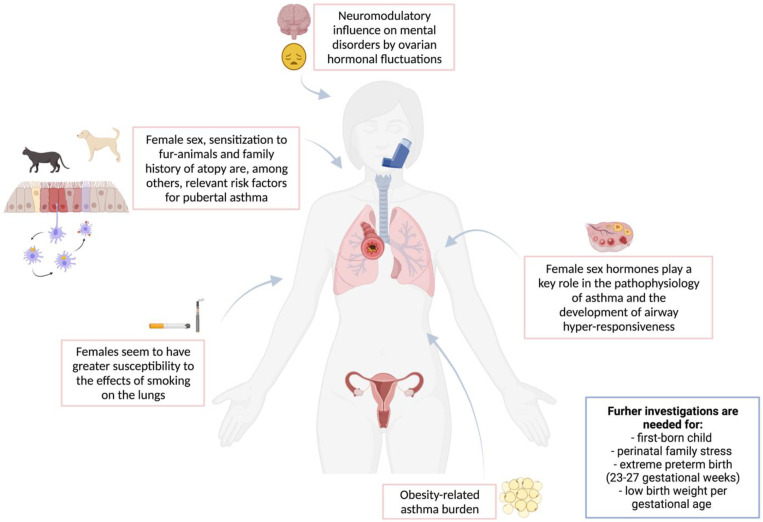
Risk factors for asthma in young female adolescents.

Figure 2 summarizes risk factors for asthma in young female adolescents.

## 5. Perimenstrual Asthma

PMA is usually defined as cyclical exacerbation of asthma symptoms during the luteal phase and/or during the first days of the menstrual cycle [144,145].

The first case of a woman with serious recurrent PMA exacerbations was described in 1931 [7]. Inhibition of ovarian function stopped the symptoms; nevertheless, with the return of hormonal function, they reappeared. These observations revealed the relevance of menstrual-cycle-related variations of sex hormones in the pathogenesis of PMA.

**Figure 3:**
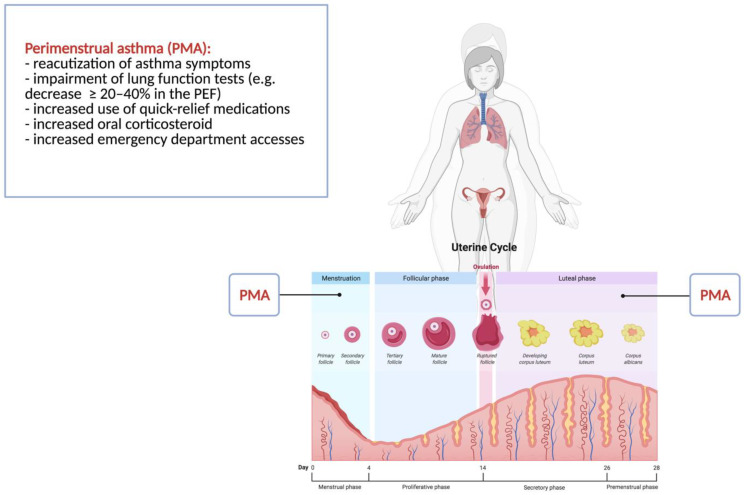
Perimenstrual asthma in young female adolescents.

The worsening of asthma is defined as a reacutization of symptoms and/or impairment of lung function tests, such as a decrease of ≥20–40% in the peak expiratory flow (PEF) [8,15,107] (see Figure 3).

A straight definition of PMA in the literature is still lacking, and discrepancies in PMA definition highly influence data recall in different studies and prevalence in different populations [146].

PMA incidence is reported to be between 19 and 40% of asthmatic women [144]. In population-based studies, asthma hospitalization rates are similar by sex in early adolescence [97,144,147,148], although they are up to three times higher in women than in men aged 20–50 years. Following menopause, the asthma incidence drops, and the return equals that of men [97,144,147,148]. The occurrence of PMA has been correlated with an increase in the number of asthma-related emergency-room visits, hospital admissions, and emergency treatment. Emergency-room admissions most commonly occur among women in the preovulatory and perimenstrual phases [13].

These data, together with abundant evidence on sex differences in asthma [147,149], support the hypothesis that hormonal status may influence asthma in women, focusing on the role of sex hormones, and particularly the impact of estrogen fluctuations at ovulation and before menstruation [147].

Perimenstrual fluctuations of sex hormones in women are considered responsible for the specific worsening of many different perimenstrual symptoms and specific inflammatory [150], autoimmune [151,152], and pain-related conditions [144], thus confirming their pathogenic role.

## 6. Perimenstrual Asthma and Sex Hormones

Various studies, including the PIAMA (Prevention and Incidence of Asthma and Mite Allergy) study, have shown that there is a gender difference in asthma that varies with age [153].

The PIAMA study [139] enlisted 4146 pregnant women and assessed 3308 of their children yearly for wheezing and asthma, using questionnaires. Males showed an increased incidence of wheezing compared to females. At the age of 8, 15.1% of the male patients and 10.8% of the female patients had asthma, suggesting that the gender difference in asthma could begin early in infancy. Males also showed more atopic symptoms, measured by specific IgE or skin-prick testing to common allergens, compared to female patients prior to adolescence [75,153,154,155,156,157]. Another study showed that phytohemagglutinin-induced mononuclear cells from males, compared to females, have remarkably increased IFN-γ, IL-5, and IL-13 in children that showed wheezing at 3 years of life. Increased rates of sensitization, total IgE levels, and blood eosinophil counts were higher in males. The disproportionate growth between lung size and airway caliber has also been detected more often in male patients [155,158]. Therefore, a more robust immune response and a decreased airway size likely contribute to increased wheezing in young males compared to females. 

Various studies have shown that hospital admissions for asthma are similar by sex in the early teenage years (10 to 13 years of age), but they are up to three times higher in females than males between 20 to 50 years. It is also reported that, after menopause, the incidence of asthma decreases balancing again with men [97,147,148].

Sex hormones are known to be effective modulators of immune responses and inflammatory-associated diseases [159,160,161]. 

Estrogens play a key role in influencing the course of various autoimmune diseases, as well as infectious processes (viral, bacterial, and others), exerting their actions through the estrogen receptor alpha and beta, as ultimately expressed by several immune cells [161].

Classical estrogen receptors (ER) ERα and ERβ are members of the superfamily of nuclear receptors. The binding of a ligand to ERα or ERβ triggers receptor activation, dimerization, and translocation from the cytoplasm to the nucleus. Here, the hormone-receptor complex recruits co-regulators and binds to estrogen receptor elements (ERE) of targeted genes, thus modulating gene transcription (genomic mechanism) [162]. More recently, researchers identified the membrane estrogen receptor GPR30 or GPER, a 7-transmembrane G-protein-coupled receptor that activates intracellular signaling cascades, including MAPK, ERK1/2, and PI3K pathways [103,163]. Thus, rapid nongenomic mechanisms through secondary messengers determine variations in cellular enzymatic pathways, ion channel, and intracellular calcium levels, and they also result in transcriptional modulation [103,164]. 

Experimental evidence suggests that ERs are involved in lung development [164]. Indeed, ERα modulates alveolar regeneration and alveolar size and number, while ERβ induces normal elastic tissue recoil through regulation of extracellular matrix [165]. In human lung tissue, ERα and ERβ are expressed in bronchial epithelial cells [103], as well as in various immune cells, including macrophages, lymphocyte, and dendritic cells [166]. It has been demonstrated that ERβ activation modulates airway inflammation and negatively regulates eosinophilic airway infiltration during asthma [167]. GPER also exerts a negative control on airway inflammation through IL-10 [168]. ERs activation in both vascular endothelial cells (especially ERα) and bronchial epithelial cells (especially ERβ) leads to nitric oxide synthesis and subsequent vasodilation or bronchodilation, respectively [103]. ERα and ERβ are expressed also in human airway smooth muscle (ASM) cells: it was observed that asthmatic subjects manifest an increased ERs expression in ASM which concerns especially ERβ subtype [169]. A murine model of asthma revealed pronounced airway fibrosis and ASM hypertrophy, leading to airway hyper-reactivity (AHR) and remodeling. Interestingly, this condition reversed with ERβ activation [167,170] and downregulation of extracellular matrix proteins [171]. Furthermore, it was observed that ERβ activation diminishes ASM thickness through the negative regulation of PDGF (platelet derived growth factor)-induced proliferation in human ASM cells [170]. According to these evidences, in a recent study on mice, it was confirmed that asthmatic phenotype was associated to airway remodeling and subsequent AHR. In that context, ERs may play a major downregulating role, in which the activation of ERβ (but not ERα) resulted in decreased remodeling and AHR [167].

Patients with asthma generally show allergic airway inflammation characterized by type-2-mediated airway inflammation, but some patients show low type-2-mediated airway inflammation with increased neutrophils concentration caused by type 1 or IL17-mediated airway inflammation [172,173]. Type 2 allergic airway inflammation starts with exposures to allergens, including dust, pollen, mammalian antigens, cockroach antigens, and others, resulting in increased production of inflammatory cytokines, such as thymic IL-4, IL-25, and IL-33. Increased concentration of these co-stimulatory cytokines results in enhanced expression of proinflammatory cytokines; IL-4, IL-5, IL-13, and IL-9 produced by TCD4+ cells helper; group-2 innate lymphoid cells (ILCs); eosinophils; basophils; mast cells; macrophages; and others. The release of these cytokines leads to increased IgE-triggered hypersensitivity to allergens, activation of airway epithelial cells, activation and infiltration of eosinophils, mucus production, and AHR. Increased secretion of IL-17A, a cytokine secreted by CD4+ Th17 cells and other cell types, also leads to increased airway inflammation and hyper-responsiveness [174]. 

Such an immune response can be modulated by hormonal milieu during different stages in life, from puberty to menopause [159,160,161]. Thus, sex-hormone fluctuations seem to play a key role in respiratory health, leading to asthma exacerbations, suggesting the need of monitoring hormonal changes in asthmatic female patients. 

During puberty, testes increase testosterone production, along with adrenal glands producing androgens, leading reproductive organs to mature and muscle and bone to grow. For what concerns females, there is an increase in the production of estrogen from the ovaries (driving thelarche and menarche), along with FSH and LH, and androgens (androstenedione and DHEA-S) from the adrenal glands. 

Hyperandrogenism may be present in some adolescents more likely suffering from polycystic ovary syndrome (PCOS), a medical condition showing a certain degree of comorbidity with asthma [175], especially in overweight and obese adolescents [176].

The androgen overproduction with puberty seems to confer protection on lung growth in both males and females, while estrogens may well have negative effects in females extending into adulthood. 

It has been observed that progesterone is the pivotal hormone in the perimenstrual phase, which is also when this specific asthma phenotype occurs [177]. Patients with PMA frequently show impairment in periodic fluctuations in serum progesterone concentrations [10,178]. Progesterone, as well as all other steroid hormones, is synthesized from pregnenolone. Progesterone is an aldosterone precursor, which, in turn, can be converted into testosterone [179]. Some data have shown that low testosterone levels can significantly impair immune responses and airway smooth-muscle reactivity, either through genomic or non-genomic mechanisms [13,180]. 

De Boer et al. [13] studied 116 males and 71 females, showing that female patients had post-bronchodilator FEV1% and FVC% significantly lower (by 8.9% and 9.1% respectively) than the same values seen in male patients from pre-/early to mid-/late puberty, as determined by breast development (differences attributed by Tanner stage). For what concerned female patients between 6 and 18 years of age, androgens correlated positively with lung function, whereas estrogens did not. Free testosterone had a favorable connection with post-bronchodilator FEV1%. On the other hand, estradiol showed negative coefficients for pre- and post-bronchodilator FEV1% and FVC%. 

Moreover, DHEAS, which works as an inhibitor of the airway smooth muscle and fibroblast proliferation, influences airway epithelial-to-mesenchymal transition and even prevents airway remodeling observed in severe asthma. All of these effects, in association with the increased levels of DHEA-S reported in male subjects during late adolescence, may explain lower symptoms in case of hyperandrogenism.

These data suggest that the high rate of asthma exacerbations in females during late adolescence is due to the negative role of estradiol; it also consolidates the benefit of androgens, which are quite low in females [13].

Studies showed reacutization of asthma symptoms, reduced peak flow rates, and increased use of quick-relief medications in nearly 40% of female patients with asthma during the pre- or perimenstrual phase of the cycle [104,105,142,181,182].

As far as the use of contraceptive measures is concerned, some studies, such as the SAPALDIA (Swiss cohort study on Air Pollution And Lung Disease in Adults) [183], showed a reduction in methacoline-induced airway hyper-responsiveness in pill users. DHEA, which is ultimately converted into androgens and estrogens, has been tested in animal studies in order to explore its asthma-related role. It happened to lower the airway eosinophils, along with IL-4 and -5 serum levels in mice fed with it [184,185]. Nebulized dehydroepiandrosterone-3-sulfate, when used as a medication in moderate-to-severe asthmatic patients seemed to improve asthma control. Surely, further studies are needed in order to evaluate the possible therapeutic use of such hormonal compounds in asthmatic patients [186].

It appears clear that sex hormones have an important role in the immune system in many autoimmune and/or allergic diseases, including asthma. Therefore, gender differences in asthma prevalence coincide with modifications in sex hormones levels, highlighting their key role in regulating PMA asthma pathogenesis. 

## 7. Perspective Strategies in Perimenstrual Asthma Treatment

To our knowledge, there are no studies specifically investigating treatment of PMA in adolescents. Moreover, the literature about the management of PMA in reproductive-age women shows contradictory results, mainly obtained from small heterogeneous cross-sectional studies [187].

### 7.1. Conventional Asthma Therapy

According to the latest iteration of the Global Initiative for Asthma (GINA), the recommended therapy for mild-to-moderate asthma in adolescents is a combination of inhaled corticosteroids and formoterol (long-acting beta2-agonist, LABA) as both a maintenance and a reliever therapy, while severe asthma requires expert assessment and specific add-on therapy [2]. Short-acting beta2-agonists (SABA) are no longer recommended as an asthma-only treatment, because they do not protect against severe exacerbations, and their overuse is associated with an increased risk of asthma deterioration and disease-related mortality [2,188]. The assessment of asthma exacerbations, even in the perimenstrual phase, requires checking for common problems, such as incorrect inhaler technique, poor adherence, or risk-taking behaviors (i.e., smoking), and information and self-management strategies should be provided [2,189]. In a randomized crossover study, 13 women with PMA received long-acting beta2-agonist (salmeterol) or placebo in the 10 days prior to menstruation and a significant complete remission of PMA was observed in 54% of women following salmeterol administration, suggesting that the conventional treatment with inhaled corticosteroids and LABA prevents PMA in most of the patients [190]. In addition to the usual strategies for asthma management, alternative treatment modalities have been investigated to aid in the reduction of perimenstrual exacerbations. Indeed, according to GINA, contraceptive pills and/or leukotriene receptors antagonists may be helpful (evidence D) [2]. 

### 7.2. Hormonal Contraception

The rationale behind the potential use of hormonal contraception (HC) to treat PMA is the suppression of endogenous sex steroids fluctuations that are considered a possible pathogenetic mechanism of perimenstrual exacerbation [191]. Furthermore, HC may treat hormonal imbalance underlying irregular menstrual cycles that are frequent in adolescents [24] and linked with an increased risk of asthma in older women [28,30]. However, the literature about the impact of HC on asthma and perimenstrual exacerbations is scarce, of low quality, and contradictory, especially for young females, because it lacks randomized trials and a clear definition of PMA.

Macsali et al. conducted a large cross-sectional survey on fertile-aged women and reported that the use of combined oral contraceptives (COCs) containing estrogens and progestogens was associated with an increased risk of asthma and wheeze in normal weight or overweight women but not in lean women [192]. Erkoçoğlu et al. investigated the effect of COC on wheezing in a sample of 487 adolescents and young women of which 40.2% were COC users and found that COC use was related to an increased risk of wheezing after adjusting for asthma and smoking [193]. In a genome-wide DNA study by Guthikonda et al., COC use in adolescents was associated with DNA methylation of a T helper 2 (Th-2) transcription factor, thus determining an increased risk of asthma [194]. 

Some studies did not exhibit any influence of HC on asthma. Tan et al. reported that COC use did not alter beta2-adrenoreceptor function of forced expiratory volume (FEV1) in 11 reproductive-aged women with stable moderate asthma [195], and a large cross-sectional study including 24% of COC users did not confirm an association with self-reported asthma [196]. Moreover, in a sample of 28 asthmatic women prospectively followed, the proportion of COC use was not different between women with and without PMA, suggesting exogenous hormonal withdrawal as a possible mechanism leading to PMA [146].

Conversely, there is evidence for a protective effect of exogenous sex steroids on asthma symptoms. A study from Tan et al. evaluated lung function in female asthmatic with spontaneous menstrual cycles or COC use observing an attenuated cyclical change in airway reactivity, as well as reduced peak expiratory flow rate variability in asthmatic patients receiving COC [197]. Moreover, in a pilot study on 13 asthmatic women, COC appear to enhance regulatory T cells, leading to better asthma control [198]. Salam et al. explored the impact of COC on a large cohort of post-pubertal young girls with and without history of asthma and found that COC use was associated with a markedly reduced prevalence of wheezing symptoms in asthmatic women, with significant trends related to duration of COC [69]. In a cross-sectional survey of the Scottish general population involving 3257 women aged 16–45 years, any HC was associated with reduced risk of current asthma and number of exacerbations with need of care [199]. Even in an Australian study involving young women, COC use and duration was associated with a decreased risk of current asthma, but did not predict asthma among users who had a history of asthma or wheeze during childhood [200]. More recently, the largest longitudinal retrospective study by Nwaru et al. based on a primary care database in United Kingdom investigated the association between HC and the risk of severe asthma exacerbations in asthmatic women of reproductive age. Previous and current use longer than 3 years of combined (estrogen/progestogen) contraceptives, but not progestogen-only contraceptives, was associated with a reduced risk of severe asthma exacerbations across all body mass index (BMI) categories [201]. Furthermore, in another population-based cohort study of 16–45-year-old women, previous and current use of HC (both combined and progestogen-only) and longer duration of use were related to a reduced risk of new onset asthma [202].

HC provides lower and more stable levels of circulating hormones, thus modulating the immune and inflammatory responses with beneficial effects in asthmatic women vulnerable to PMA [144,198,203]. A valid hypothesis to better control PMA might be the reduction of the hormone-free interval (HFI) when HC is considered [144]. Indeed, shorter HFI or extended/flexible regimens of HC reduce hormone-withdrawal associated symptoms and systemic inflammation, provide more powerful ovarian suppression with a decreased production of endogenous hormones, and enhance adherence to treatment [204,205]. A limitation of previous large studies based on primary care databases is the extensive missing data about the subtypes of HC analyzed, while other studies with smaller sample sizes do not specify the formulations of the administered COC. Therefore, prospective controlled studies comparing different regimens of HC, as well as molecules with peculiar biochemical characteristics, are needed to test this strategy. 

Another risk factor for asthma that could potentially benefit from HC is anemia, since anemic children resulted in being more susceptible to asthmatic attacks [206]. Heavy menstrual bleeding (HMB) is a common gynecological condition in female adolescents and a major cause of anemia [207]. Thus, the use of COC, especially with shorter hormone-free interval or in extended regimens significantly reduces the duration and severity of menstrual bleeding with potential benefits for asthma [144,208]. In this perspective, also iron supplementation could exert a protective effect on the respiratory system, considering the evidence that iron deficiency is more common in asthmatic women [209]. Another interesting finding deserving more research is the evidence that adolescent endometriosis, a chronic inflammatory condition associated with dysmenorrhea and HMB, is comorbid with a history of asthma [210]. Indeed, women with PMA more often have dysmenorrhea, premenstrual syndrome, shorter menstrual cycles, and longer menstrual bleeding [211]; hence, they may benefit from HC use [212]. 

### 7.3. Estrogens, Progestogens, and Androgens

A few small studies have described alternative hormonal treatments for PMA, obtaining conflicting results. The administration of exogenous estradiol in 14 asthmatic women with and without PMA improved asthma symptoms and dyspnea index score [8]. Recently, in a large cross-sectional survey including pre- and postmenopausal women, it was highlighted that elevated estradiol and free testosterone levels were associated with reduced risk of current asthma in obese women. Even if inference seems unlikely because of the study design, estradiol may have variable effects on immune responses depending on its concentration, timing, and duration of exposure [213]. 

Rubio et al. found that 80% of 30 asthmatic women had at least one hormone (either progesterone, estradiol, or cortisol) out of range, with the most common abnormality being the reduced progesterone on day 21 of menstrual cycle, especially in women with PMA. However, in 55% of women with PMA, no relationships between decreased progesterone and perimenstrual asthma could be demonstrated [10]. By contrast, Tan et al. showed normal luteal increases in serum progesterone (and estradiol) in PMA women [214]. As observed in other catamenial conditions, for example, migraine headache [215], it is likely that the amount of hormonal fluctuations more than the absolute values play a role in the manifestation of PMA.

The synthetic progestogen medroxyprogesterone acetate (MPA) has been investigated as a possible treatment for PMA. In 1988, three women with severe unresponsive PMA obtained improvement after continuous administration of MPA [216]. In a subsequent study, Tan et al. found a paradoxical downregulation of beta2-adrenoreceptors by MPA in seven asthmatic women [217]. More recently, MPA resulted in immunosuppressive functions through inhibition of Th-1, Th-2, and Th-17 responses [11]. To our knowledge, no comparative data are available taking into account the pharmacological characteristics of progestogens in the treatment of PMA.

Androgens, especially DHEA and DHEA-S, are associated with better lung function as a result of inhibition of airway hyper-reactivity, eosinophils, serum IL-5 production, leukotriene synthesis, and proliferation of airway smooth-muscle cells and fibroblasts, thus preventing airway remodeling typical of severe asthma [12,218,219]. Consequently, the androgen surge with puberty is supposed to confer protective effects on lung growth. Moreover, in late adolescence, the presence of more asthma symptoms in females compared to males either reinforces the benefit of androgens (lower in females) or supports a negative role for estrogens [13]. DHEA-S levels are reduced in patients with asthma [220] and decrease in a dose-dependent manner with the use of inhaled corticosteroids, allowing us to consider DHEA replacement therapy as a possible strategy [12]. DHEA-S has already been investigated as a potential therapeutic agent for asthma in a randomized placebo-controlled trial, where a nebulized formulation of DHEA-S improved asthma control in moderate-to-severe asthmatics [186]. It is also plausible to consider a trial investigating androgen treatment in adolescents with low androgen levels and poorly controlled asthma, with the aim of improving lung function [13].

### 7.4. Leukotriene Receptor Antagonists

Leukotriene receptor antagonists exert a combined anti-inflammatory and bronchodilating effect [14] and are considered an adjuvant therapy for asthma exacerbations, according to a GINA statement [2]. Women with PMA exhibit significantly higher levels of serum leukotrienes in the premenstrual phase than in preovulatory, while no differences in serum leukotriene concentrations occur in asthmatic women without PMA [15]. Hence, leukotriene receptor antagonists have been proposed as a specific treatment for PMA. Nakasato et al. observed a significant improvement of asthmatic symptoms and peak expiratory flow rate in women with severe PMA after administration of the leukotriene receptor antagonist pranlukast [15]. Pasaoglu et al. obtained similar results in women with mild PMA who received montelukast, observing the absence of this beneficial effect in asthmatic women without PMA [221]. Conversely, Pereira-Vega et al. analyzed serum leukotriene variations in women with and without moderate PMA, finding no differences in leukotriene levels between the preovulatory and premenstrual phases in these groups, and, therefore, not supporting an involvement of leukotrienes in the pathogenesis of moderate perimenstrual asthma [14]. 

### 7.5. Microbiota

Over the last few years, gut microbiota emerged as a possible contributor to the development of asthma in children and adults [222], with a focus on intrauterine and neonatal factors that could affect subsequent respiratory and atopic symptoms [16]. Recent advances in understanding microbial ecology highlight the complex interactions between environmental microbes, respiratory microbial communities, and gut microbiota through the “gut–lung axis” [17]. Thus, deeper exploration of this innovative field may raise the possibility of new microbial-directed treatment strategies with the aim of improving or preventing asthma and its exacerbations. 

### 7.6. Vitamin D

Vitamin D is a secosteroid hormone with multi-organ targets: it has known hormonal, metabolic, and immunomodulatory functions virtually in every organ system, including the musculoskeletal, cardiovascular [223,224,225], immune [226,227], and reproductive system [228,229,230,231]. It is a fat-soluble nutrient which also plays an important role in immune regulation and respiratory diseases and infections [232,233]. Serum 25-OH vitamin D is the main indicator of total vitamin-D status, as it reflects vitamin D intake from dietary sources, as well as sun exposure, thus promoting photosynthesis in the skin; it also accounts for vitamin D adaptation from adipose stores in the liver [233]. Even if there are no guidelines on optimal serum levels, vitamin D deficiency is usually defined as a 25-OH vitamin D level below 50 nmol/L (20 ng/mL) [223,234]. It has been proposed that lifestyle modernization and Westernization have led to vitamin D deficiency among the world’s population, related to higher rates of sedentary lifestyle and time spent indoors, away from sun exposure [235,236]. 

The role of vitamin D in asthma remains not yet well understood. However, some cross-sectional investigations have suggested a possible link between asthma and vitamin D [236,237]. Clinical data have indicated that a reduced serum 25OH-vitamin D level was associated with increased prevalence, hospitalization, and increased emergency visits, along with declining lung function and increased airway hyper-responsiveness in asthmatic patients [236,238]. In addition, a protective influence of vitamin D supplementation among asthmatic patients has recently been observed [239,240,241]. Moreover, increased vitamin D intake during pregnancy has an influence on asthma in children and adults [242,243]. Vitamin D has also been observed to play a role in asthma exacerbations. Several cross-sectional and longitudinal studies have reported that increased vitamin D levels correlated with reduced asthma exacerbations and reduced emergency department admissions [223,244]. In contrast, serum vitamin D concentration was significantly decreased in asthma patients compared with the control group [245], and asthma patients with vitamin D insufficiency were more likely to have exacerbations [223,246].

As reported, vitamin D supplementation when deficient or insufficient has a protective role against asthma and its exacerbations [223,239,240,241,247]. All trials concerning vitamin D supplementation [248,249,250,251,252,253] have indicated that providing vitamin D3 could improve most somatic and affective perimenstrual syndrome (PMS) severity, including PMA [230]. Nevertheless, an optimal dose of vitamin D3 supplementation and also best treatment length for improving symptoms have not been established [230].

There are many data confirming the role of inflammation in PMS [248]. Azizieh et al. emphasized a possible role of pro-inflammatory cytokines, such as IL-8 and TNF-α, as contributing factors to PMS symptoms [254]. Vitamin D, as an anti-inflammatory agent, could act by increasing anti-inflammatory cytokines, such as transforming growth factor β, and decreasing inflammatory cytokines, such as tumor necrosis factor α, IL-10, and IL-12 [248]. Apparently, the effect of vitamin D on cytokines is mediated by the action of 1α,25-dihydroxy vitamin D3, which acts by inhibiting IL-12 production in activated macrophages [248,252]. According to Johnson et al., there is a very strong correlation between total symptom score in women with PMS and IL-10 and IL-12 levels [255]. Therefore, the results obtained in the study by Heidari et al. indicate beneficial effects of fortnightly 50,000 IU vitamin D3 supplementation on symptoms and inflammatory and antioxidant markers in vitamin-D-deficient students with PMS and PMA [248]. In addition, no adverse effects of supplementation were reported in participants [248]. 

## 8. Conclusions

The role of female sex hormones in the clinical expression of asthma across the menstrual cycle is crucial. Asthma exacerbations begin more often during the preovulatory period and ovulation; the associated fluctuations of sex hormones may trigger asthmatic crisis in susceptible women.

PMA is a difficult-to-treat asthma phenotype in which conventional asthma therapies are not always effective. 

New preventive COC strategies providing stabilization of estrogens and progesterone/progestins levels by also reducing the HFI or the number of bleeding episodes may be considered. Beneficial effects of other hormonal treatments, including estrogens, progestogens and androgens, and leukotriene receptor antagonists have been described. An explorative approach using microbial-directed therapy may be also investigated. At present, the management of PMA remains a challenge for pediatricians and gynecologists. Further studies focused on young females are mandatory to promote adolescent health.

## Figures and Tables

**Table 1:** Normal values of sex hormones in different phases of the menstrual cycle.

	Early Follicular Phase	Preovulatory Phase	Midluteal Phase
Estradiol (pg/mL)	40–50	250–380	100–250
Progesterone (ng/mL)	<1		10–35
Androstenedione (ng/mL)	2.2	2.7	2.6
Testosterone (nmol/L)	0.96	1.27	0.91

## Data Availability

Not applicable.
